# Serum sex hormone-binding globulin levels are reduced and inversely associated with intrahepatic lipid content and saturated fatty acid fraction in adult patients with glycogen storage disease type 1a

**DOI:** 10.1007/s40618-022-01753-2

**Published:** 2022-02-07

**Authors:** P. I. H. G. Simons, O. Valkenburg, I. Telgenkamp, K. M. van der Waaij, D. M. de Groot, P. Veeraiah, J. A. P. Bons, T. G. J. Derks, C. G. Schalkwijk, V. B. Schrauwen-Hinderling, C. D. A. Stehouwer, M. C. G. J. Brouwers

**Affiliations:** 1 Division of Endocrinology and Metabolic Diseases, Department of Internal Medicine, Maastricht University Medical Centre, PO Box 5800, 6202 AZ Maastricht, The Netherlands; 2 Laboratory for Metabolism and Vascular Medicine, Maastricht University, Maastricht, The Netherlands; 3 CARIM School for Cardiovascular Diseases, Maastricht University, Maastricht, The Netherlands; 4 Department of Reproductive Medicine, Maastricht University Medical Centre, Maastricht, The Netherlands; 5 Department of Nutrition and Movement Sciences, Maastricht University, Maastricht, The Netherlands; 6 Department of Radiology and Nuclear Medicine, Maastricht University, Maastricht, The Netherlands; 7 Central Diagnostic Laboratory, Maastricht University Medical Centre, Maastricht, The Netherlands; 8 Section of Metabolic Diseases, Beatrix Children’s Hospital, University Medical Centre Groningen, University of Groningen, Groningen, The Netherlands; 9 Division of General Internal Medicine, Department of Internal Medicine, Maastricht University Medical Centre, Maastricht, The Netherlands

**Keywords:** Glycogen storage disease type 1a, De novo lipogenesis, Intrahepatic lipid content, Sex hormone-binding globulin

## Abstract

**Purpose:**

De novo lipogenesis has been inversely associated with serum sex hormone-binding globulin (SHBG) levels. However, the directionality of this association has remained uncertain. We, therefore, studied individuals with glycogen storage disease type 1a (GSD1a), who are characterized by a genetic defect in glucose-6-phosphatase resulting in increased rates of de novo lipogenesis, to assess the downstream effect on serum SHBG levels.

**Methods:**

A case–control study comparing serum SHBG levels in patients with GSD1a (*n* = 10) and controls matched for age, sex, and BMI (*n* = 10). Intrahepatic lipid content and saturated fatty acid fraction were quantified by proton magnetic resonance spectroscopy.

**Results:**

Serum SHBG levels were statistically significantly lower in patients with GSD1a compared to the controls (*p* = 0.041), while intrahepatic lipid content and intrahepatic saturated fatty acid fraction—a marker of de novo lipogenesis—were significantly higher in patients with GSD1a (*p* = 0.001 and *p* = 0.019, respectively). In addition, there was a statistically significant, inverse association of intrahepatic lipid content and saturated fatty acid fraction with serum SHBG levels in patients and controls combined (*β*: − 0.28, 95% CI: − 0.47;− 0.09 and *β*: − 0.02, 95% CI: − 0.04;− 0.01, respectively).

**Conclusion:**

Patients with GSD1a, who are characterized by genetically determined higher rates of de novo lipogenesis, have lower serum SHBG levels than controls.

## Introduction

It has long been thought that sex hormone-binding globulin (SHBG) acts only as a simple carrier protein that regulates the bioavailable fraction of testosterone and other sex hormones [1]. In the past decades, however, serum SHBG has also been inversely associated with several metabolic disorders, including obesity, non-alcoholic fatty liver disease, and type 2 diabetes [2–4]. Even more recently, SHBG has been identified as a hepatokine that protects from type 2 diabetes [5, 6]. These observations stress the need for a better understanding of the regulation of serum SHBG levels in humans.

In vitro and animal studies have shown that carbohydrate-induced de novo lipogenesis is one of the mechanisms involved in the downregulation of SHBG levels [7]. We have recently extrapolated these findings to humans, by showing that de novo lipogenesis, measured with stable isotopes, is inversely associated with serum SHBG levels [8]. However, given the observational nature of that study, we were unable to assess whether the effect of de novo lipogenesis on serum SHBG is causal. This is of importance, since previous in vitro and animal studies have shown that the association between de novo lipogenesis and SHBG appears to be bidirectional, i.e. serum SHBG may also directly reduce the rates of de novo lipogenesis [9, 10].

Monogenetic disorders that affect de novo lipogenesis can be used to unravel whether there is a causal effect of de novo lipogenesis on serum SHBG, in humans. Glycogen storage disease type 1a (GSD1a) is an inborn error of metabolism, caused by a mutation in the *G6PC* gene encoding glucose-6-phosphatase [11]. As a consequence, there is an intrahepatic surplus of glucose-6-phoshate that can serve as a substrate for glycolysis and de novo lipogenesis. Previous studies have shown that patients with GSD1a are indeed characterized by higher rates of de novo lipogenesis and intrahepatic saturated fatty acid (SFA) fraction, the product of de novo lipogenesis [12, 13].

The aim of this study was, therefore, to examine serum SHBG levels in patients with GSD1a and controls matched for age, sex, and BMI, and to study the relationship of intrahepatic lipid (IHL) and SFA content with serum SHBG.

## Methods

### Study design

For this case–control study, we recruited homozygous carriers of a mutation in the gene encoding glucose-6-phosphatase (*G6PC*), causing GSD1a, from outpatient metabolic clinics in the Netherlands and Belgium. Cases were matched to controls based on factors that are known to affect serum SHBG levels, i.e. age, sex, and BMI [14, 15]. To accomplish an adequate matching, we retrieved data for controls from (1) the effects of fructose restriction on liver steatosis [FRUITLESS] study [16], (2) the aldolase B deficiency study [17], and (3) prospective recruitment through local advertisement. All study protocols were similar, with the exception of differences in in- and exclusion criteria. The rationale and design of the FRUITLESS and aldolase B deficiency studies have been published previously. In short, the FRUITLESS study was originally conducted to assess the effects of fructose restriction on IHL content [16]. Participants were included if they had a fatty liver index ≥ 60, and excluded in case of a history of liver disease, excessive alcohol consumption, change in weight or physical activity 3 months prior to participation, use of glucose-lowering drugs, recent illness, pregnancy and/or lactation [16]. For the current study, only data from baseline measurements were used. The aldolase B deficiency case–control study was originally conducted to compare IHL content in patients with hereditary fructose intolerance and controls. Only data from controls was used in this study [17]. All studies were performed according to the Declaration of Helsinki and approved by the Medical Ethical Committee of Maastricht University Medical Centre [18]. All participants gave written informed consent prior to participation.

All participants had to be at least 18 years of age and were excluded from participation if they had any contraindications for magnetic resonance imaging (MRI) or were unable to give informed consent. Furthermore, as oestrogen-containing medication, which substantially affects serum SHBG levels, are relatively contra-indicated in GSD1a [19–21], controls were excluded if they used oestrogen-containing medication. All controls were asked to visit the metabolic ward after an overnight fast. Since patients with GSD1a develop hypoglycaemia and lactic acidosis upon prolonged fasting, they are treated with either (modified) uncooked cornstarch at night or continuous nocturnal feeding with dextrose [11]. They were, therefore, asked to visit the metabolic ward before having breakfast.

### Measurements

For all participants, anthropometrics and quantification of IHL content by proton magnetic resonance spectroscopy (^1^H-MRS) using a 3T clinical MR scanner (Achieva 3T-X, Philips Healthcare, Best, the Netherlands), were performed as described previously [17]. In a subset of GSD1a patients and controls, the hepatic SFA fraction (expressed as the ratio SFA:IHL * 100%) was quantified with a newly developed ^1^H-MRS method. We previously showed that this fraction correlates well with de novo lipogenesis assessed by stable isotopes [13]. Serum uric acid, total cholesterol, HDL cholesterol, and triglycerides were measured with an enzymatic colorimetric assay (Cobas 8000 instrument, Roche Diagnostics, Mannheim, Germany). Serum glucose was measured with an enzymatic spectrophotometric assay in patients with GSD1a and a subset of the controls (Cobas 8000 instrument, Roche Diagnostics, Mannheim, Germany). In the controls originating from the aldolase B deficiency and FRUITLESS studies, serum glucose was measured with YSI2300 STAT Plus Glucose Lactate Analyzer (YSI, Yellow Springs, OH, USA) [16, 17]. Serum insulin and SHBG were measured with a chemiluminescent immunometric assay in all participants (Immulite XPi instrument, Siemens Healthcare Diagnostics, New Orleans, LA, USA). Serum SHBG measurements demonstrated an analytical variability of 5–7%, with an intra-individual biological variability of 9% [22]. In one patient with GSD1a, blood was drawn directly after the consumption of food, and hence this individual was not included in the analyses of fasting-sensitive measures (i.e., serum lipids, glucose and insulin).

### Statistical analyses

Continuous data are presented as median (interquartile range) and categorical data are presented as frequencies. Continuous variables were compared between GSD1a patients and controls by means of Mann–Whitney *U* test. Univariate regression analyses were performed to study the association of BMI, serum insulin, IHL content and SFA fraction with serum SHBG levels. The univariate regression analyses were additionally stratified by condition (i.e., GSD1a or controls). All results were considered statistically significant at *p* < 0.05. All statistical analyses were performed using IBM Statistical Package of Social Science (SPSS) version 25.0 for Windows (IBM Corp., Armonk, N.Y., USA).

## Results

### Population characteristics

The general characteristics of the patients with GSD1a (*n* = 10) and matched controls (*n* = 10; *n* = 5 from FRUITLESS, *n* = 3 from the aldolase B deficiency study, and *n* = 2 prospectively recruited) are shown in Table 1. The majority of the study population was female (70%), young (median age: 31.0, IQR: 20.5–47.0 years) and overweight (median BMI: 25.5, IQR: 23.6–29.7 kg/m^2^). Age, sex distribution and BMI were, by design, comparable between GSD1a patients and controls (Table 1). Serum triglycerides were statistically significantly higher in individuals with GSD1a, while HDL-cholesterol was significantly lower in patients with GSD1a compared to controls (Table 1).

**Table 1:** Characteristics of controls and patients with glycogen storage disease type 1a (GSD1a)

	Control (*n* = 10)	GSD1a (*n* = 10)
Sex (male/female), *n*/*n*	3/7	3/7
Age, years	32.5 (20.0–56.3)	29.5 (21.5–47.0)
BMI, kg/m^2^	26.0 (22.2–31.5)	25.5 (24.5–28.5)
Waist circumference, cm	93.6 (80.5–110.0)	93.5 (82.8–100.4)
Systolic blood pressure, mmHg	129 (112–138)	125 (105–134)
Diastolic blood pressure, mmHg	79 (65–84)	76 (64–81)
Glucose, mmol/l^#^	4.5 (4.2–5.0)	4.4 (3.4–4.8)
Insulin, pmol/l	28.7 (23.0–67.2)	16.5 (10.0–26.6)*
Uric acid, mmol/l	0.34 (0.30–0.35)	0.37 (0.26–0.47)
Total cholesterol, mmol/l	5.1 (4.1–5.4)	5.5 (4.8–6.9)
HDL-cholesterol, mmol/l	1.2 (1.1–1.5)	0.9 (0.7–1.0)*
Triglycerides, mmol/l	1.3 (1.0–1.6)	4.9 (3.7–6.3)*
Alcohol consumption (units/week)	0.2 (0.0–5.0)	0.0 (0.0–0.3)

Unless otherwise noted, data are expressed as median (interquartile range)

*BMI* body mass index, *GSD1a* glycogen storage disease type 1a, *HDL* high density lipoprotein, *LDL* low density lipoprotein

*p < 0.05 compared to controls, analysed with Mann–Whitney *U* test

^#^Serum glucose was measured with enzymatic spectrophotometric assay in GSD1a and with enzymatic spectrophotometric assay YSI2300

STAT Plus Glucose Lactate Analyzer in controls, see “Methods” section

**Fig. 1:**
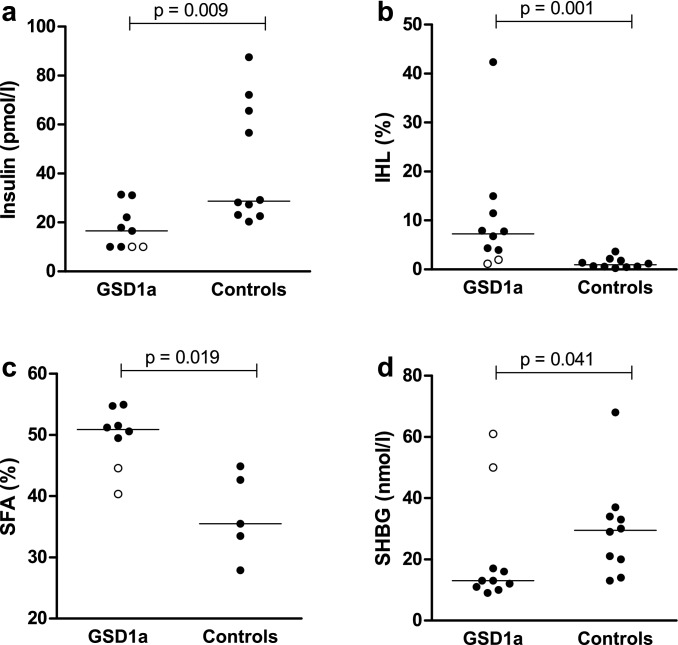
Serum insulin (**A**), intrahepatic lipid (IHL) content (**B**), saturated fatty acid (SFA) fraction (**C**) and serum sex hormone-binding globulin (SHBG) (**D**) in patients with glycogen storage disease type 1a (GSD1a) and controls matched for age, sex, and BMI. Data expressed as individual values with median. Open circles represent GSD1a patients with exceptionally high serum SHBG levels. Differences between the groups were analysed with a Mann–Whitney *U* test. Of note, fasting serum insulin was unavailable in one GSD1a patient, and SFA fraction was only measured in a subset of cases (*n* = 8) and controls (*n* = 5) (see “Methods” section)

Patients with GSD1a had statistically significantly lower levels of serum insulin compared to controls (*p* = 0.009) (Fig. 1, panel A), while the IHL content and SFA fraction in patients with GSD1a were statistically significantly higher compared to controls (*p* = 0.001 and *p* = 0.019, respectively) (Fig. 1, panel B and C, respectively). Serum SHBG levels were statistically significantly lower in GSD1a patients compared to the controls (13.0 nmol/l [IQR: 10.8–25.3] versus 29.5 nmol/l [IQR: 18.5–34.8], respectively; *p* = 0.041; Fig. 1, panel D). As one male patient used pregnyl (i.e., human chorionic gonadotropin), which may potentially influence serum SHBG levels [23], we repeated the analyses after exclusion of this patient and the matched control, which did not affect the results (*p* = 0.050).

Strikingly, two female patients with GSD1a were found to have high serum SHBG levels relative to the other (female) GSD1a patients, and were notable outliers (Fig. 1, panel D, open circles). Of interest, these two patients also seemed metabolically healthier, as indicated by relatively low serum insulin levels, IHL content, and SFA fraction compared to the values seen for the other patients with GSD1a (Fig. 1, panel A, B, and C; open circles), whereas serum triglycerides and urate did not appear to differ (data not shown).

### Determinants of serum SHBG

**Fig. 2:**
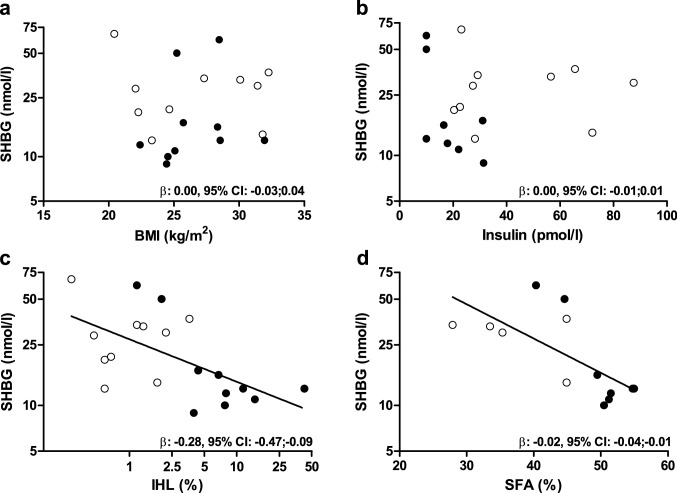
Association of body mass index (BMI) (**A**) serum insulin (**B**) intrahepatic lipid (IHL) content (**C**), and saturated fatty acid (SFA) fraction (**D**) with serum sex hormone-binding globulin (SHBG) levels stratified by glycogen storage disease type 1a (closed circles) and controls matched for age, sex, and BMI (open circles). Regression coefficients (*β*) and 95% confidence intervals (CI) are reported for the associations in the two groups combined. Of note, fasting serum insulin was unavailable in one GSD1a patient, and SFA was only measured in a subset of patients (*n* = 8) and controls (*n* = 5) (see “Methods” section)

In the overall population there was no statistically significant association between BMI or insulin with serum SHBG levels (*β*: 0.00, 95% CI: − 0.03;0.04 and *β*: 0.00, 95% CI: − 0.01;0.01, respectively), or after stratification by condition in the GSD1a group (*β*: 0.02, 95% CI: − 0.06;0.10 and *β*: − 0.02, 95% CI: − 0.04;0.01, respectively), or in controls (β: -0.01, 95% CI: − 0.04;0.03 and *β*: 0.00, 95% CI: − 0.01;0.01, respectively) (Fig. 2, panel A and B, respectively). In contrast, there was a statistically significant, inverse association between IHL content and serum SHBG levels in the overall population (*β*: − 0.28, 95% CI: − 0.47;− 0.09, Fig. 2, panel C). After stratification for condition, the association remained statistically significant only in patients with GSD1a (GSD1a: *β*: − 0.47, 95% CI: − 0.83;− 0.10, controls: *β*: − 0.07, 95% CI: − 0.57;0.44). The SFA fraction was also statistically significantly inversely associated with lower serum SHBG levels in the overall population (*β*: − 0.02, 95% CI: − 0.04;− 0.01; Fig. 2, panel D). Stratified analyses showed a significant, inverse association in GSD1a but not in the controls (*β*: − 0.06, 95% CI: − 0.08;− 0.03 and *β*: − 0.01, 95% CI: − 0.05;0.03, respectively).

## Discussion

This study shows that serum SHBG levels are statistically significantly lower in adult patients with GSD1a when compared to controls matched for age, sex, and BMI. In addition, serum SHBG levels varied noticeably within the GSD1a group and appeared to correspond with the variation of other metabolic variables, i.e., IHL content, SFA fraction and serum insulin levels. Indeed, there was a statistically significant, inverse association of IHL content and SFA fraction with serum SHBG levels in patients with GSD1a and controls combined.

The current findings support and elaborate on previous experimental and observational studies. In vitro studies have shown that monosaccharide-induced de novo lipogenesis downregulates hepatocyte nuclear factor 4 alpha, thereby decreasing SHBG synthesis in HepG2 cells [7]. In addition, incubation of HepG2 cells with palmitate, the product of de novo lipogenesis, was also found to decrease SHBG levels [7]. Although we have previously extrapolated these in vitro findings to humans by showing that de novo lipogenesis, assessed with stable isotopes, was inversely associated with serum SHBG levels, we could not assess the direction of the association [8]. This is of relevance, since in vitro experiments have also shown that SHBG can affect de novo lipogenesis [9]. By studying patients with GSD1a, who are characterized by a primary genetic defect resulting in high intrahepatic glucose-6-phosphate levels and, consequently, higher rates of de novo lipogenesis [11, 12, 24], our current observations support the concept that de novo lipogenesis results in lower serum SHBG levels in humans. This conclusion, however, deserves some caution given the small, predominantly female population that was studied, which limits the generalisability of the our findings. Furthermore, our observations do not exclude the reverse, i.e., SHBG affects de novo lipogenesis in humans.

The current findings are in line with large population studies that assessed the effect of a common variant in the glucokinase regulatory protein gene (*GCKR*), which also results in higher intrahepatic glucose-6-phosphate levels and rates of de novo lipogenesis, albeit by a different mechanism. Similar to patients with GSD1a, individuals carrying the *GCKR* minor allele are characterized by lower fasting glucose and insulin levels, higher serum triglycerides, higher rates of de novo lipogenesis and a higher IHL content [25–29]. Previous genome-wide association studies have reported that the *GCKR* minor allele is also associated with lower serum SHBG levels [5, 30].

Besides a better knowledge on the causal role of de novo lipogenesis on serum SHBG in humans, the results of this study may also have several clinical implications for patients with GSD1a. First, as recent Mendelian randomization studies have shown that SHBG is causal in the pathogenesis of polycystic ovary syndrome (PCOS), the low serum SHBG levels may contribute to the higher prevalence of PCOS that has been observed in GSD1a [31, 32]. Second, we noted that two female patients with GSD1a with relatively high levels of serum SHBG were also characterized by a better metabolic control with respect to IHL content and SFA fraction. Serum SHBG could, therefore, serve as a biomarker of metabolic control in patients with GSD1a. In comparison to current measures of metabolic control (e.g., serum triglycerides), serum SHBG has several advantages. The half-life of serum SHBG is relatively long (7 days) and can, therefore, reflect metabolic control over the past days [33]. In addition, serum SHBG levels are not directly affected by the nutritional state, i.e., fasted or fed, in contrast to serum triglycerides. Further studies are needed to assess whether intra-individual variations in serum SHBG adequately reflect changes in metabolic control in GSD1a patients.

This study has several strengths and limitations. The use of GSD1a as a model for higher rates of de novo lipogenesis is a unique approach to study the direct effects of de novo lipogenesis on serum SHBG levels in an observational setting. However, the rare nature of this disease (prevalence ~ 1 in 100,000 births [34]) results in small numbers and, consequently, low statistical power, in particular for the stratified univariate analyses. In addition, as a result of the small sample size, we were unable to explore the associations in men and women separately, or adjust for potential confounders. We, therefore, decided to match the GSD1a patients with controls based on factors that are known to have a substantial effect on serum SHBG levels. Despite adequate matching, however, it cannot be excluded that there may be residual confounding. Another limitation of this study is that we were unable to account for phase of menstrual cycle or endogenous sex hormone levels. Nevertheless, the phase of menstrual cycle is likely to have scattering effects, as de novo lipogenesis varies throughout the menstrual cycle, while serum SHBG levels remain constant [35, 36]. Despite this scattering effect, we did observe a statistically significant difference in serum SHBG levels between GSD1a patients and controls. A possible effect of endogenous oestrogens or androgens on de novo lipogenesis and serum SHBG deserves further investigation. Furthermore, as a result of the extensive matching, data for healthy controls had to be retrieved from several studies. The in- and exclusion criteria varied between the studies, and, consequently, controls originating from the FRUITLESS study are metabolically less healthy when compared to the general population [16]. This could explain the relatively low serum SHBG levels in the control group, and, hence, could have mitigated the difference in serum SHBG levels between GSD1a and controls. Finally, because of the extreme phenotype of GSD1a, there may have been pleiotropic effects that have contributed to the current findings. For instance, given the fasting intolerance in GSD1a, all measures in patients with GSD1a were conducted after (modified) uncooked cornstarch at night or continuous nocturnal feeding with dextrose (but before breakfast). This may have affected some of the fasting-sensitive outcome measures including serum triglycerides, insulin and glucose. Of note, previous studies have shown that a recent meal only mildly affects IHL content [37]. The IHL content in GSD1a patients in the current study was much higher than what can be expected from a recent meal. Furthermore, previous studies have shown that serum SHBG levels are not affected by a recent meal [38].

In conclusion, in the present study we found that patients with GSD1a, who are characterized by genetically-determined higher rates of de novo lipogenesis, have statistically significantly lower levels of serum SHBG than controls.

## Data Availability

The data sets and code generated during and/or analysed during the current study are not publicly available but are available from the corresponding author on reasonable request.
